# Focused Ultrasound Thalamotomy for the Treatment of Essential Tremor: A 2-Year Outcome Study of Chinese People

**DOI:** 10.3389/fnagi.2021.697029

**Published:** 2021-07-14

**Authors:** Peihan Wu, Wei Lin, Kun Hong Li, Hui-Chin Lai, Ming-Tsung Lee, Kevin Wen-Kai Tsai, Pai-Yi Chiu, Wei-Chieh Chang, Cheng-Yu Wei, Takaomi Taira

**Affiliations:** ^1^Department of Neurology, Chang Bing Show Chwan Memorial Hospital, Changhua County, Taiwan; ^2^MR-Guided Focused Ultrasound Center, Chang Bing Show Chwan Memorial Hospital, Changhua County, Taiwan; ^3^Research Assistant Center, Show Chwan Memorial Hospital, Changhua, Taiwan; ^4^Department of Nursing, Hungkuang University, Taichung, Taiwan; ^5^InSightec Ltd., Tirat Carmel, Israel; ^6^Department of Neurology, Show Chwan Memorial Hospital, Changhua, Taiwan; ^7^Department of Neurosurgery, Chang Bing Show Chwan Memorial Hospital, Changhua County, Taiwan; ^8^Department of Exercise and Health Promotion, College of Kinesiology and Health, Chinese Culture University, Taipei, Taiwan; ^9^Department of Neurosurgery, Tokyo Women's Medical University, Tokyo, Japan

**Keywords:** MR-guided focused ultrasound, focused ultrasound, essential tremor, thalamotomy, functional neurosurgery

## Abstract

**Background:** Essential tremor (ET) is a common movement disorder among elderly individuals worldwide and is occasionally associated with a high risk for mild cognitive impairment and dementia. This retrospective study aimed to determine the clinical outcome of unilateral magnetic resonance-guided focused ultrasound (MRgFUS) thalamotomy in Chinese patients with ET.

**Methods:** In total, 31 male and 17 female patients with drug-refractory ET were enrolled in this research study from January 2017 to September 2019. The severity of tremor and disability were assessed using the Clinical Rating Scale for Tremor (CRST) within a 2-year follow-up period.

**Results:** The mean age of the participants was 59.14 ± 13.5 years. The mean skull density ratio (SDR) was 0.5 ± 0.1. The mean highest temperature was 57.0 ± 2.4°C. The mean number of sonications was 10.0 ± 2.6. The average maximum energy was 19,710.5 ± 8,624.9 J. The total CRST scores and sub-scores after MRgFUS thalamotomy significantly reduced during each follow-up (*p* < 0.001). All but four (8.3%) of the patients had reversible adverse events (AEs) after the procedure.

**Conclusions:** MRgFUS had sustained clinical efficacy 2 years after treatment for intractable ET. Only few patients presented with thalamotomy-related AEs including numbness, weakness, and ataxia for an extended period. Most Chinese patients were treated safely and effectively despite their low SDR.

## Introduction

Essential tremor (ET) is a common movement disorder worldwide, and it affects about 1% of the overall population and 4–5% of elderly individuals (≥65 years old) (Louis and Ferreira, 2010). In a survey conducted in Beijing, the prevalence of ET among people aged ≥55 years is 3.29% (Sun et al., 2020). The condition is characterized by stereotypic tremors at a frequency of 8–12 Hz, and tremors can severely affect daily living. Further, it is occasionally associated with a high risk of mild cognitive impairment and dementia (Louis et al., 2019). Although medical treatment is initially effective, tremors are rarely suppressed with time (Findley et al., 1985; Koller and Vetere-Overfield, 1989; Diaz and Louis, 2010).

Traditionally, the choice of treatment other than drugs includes either thalamotomy, which involves ablation of the ventral intermediate nucleus (VIM) of the thalamus, or thalamic deep brain stimulation (DBS). The latter is an invasive procedure, which includes the insertion of a device inside the brain. Although it has been the standard treatment for medically intractable ET due to its safety and efficacy for long-term tremor control, adverse effects such as hemorrhage, infection, and hardware-related failures are not unusual (Blomstedt and Hariz, 2005; Engel et al., 2018). Further, DBS requires repetitive sittings with increasing expenditures.

For over 50 years, ablative treatment has evolved from invasive radiofrequency to noninvasive gamma knife radiation to magnetic resonance-guided focused ultrasound (MRgFUS). In 2016, the United States Food and Drug Administration (FDA) had approved MRgFUS for the treatment of ET (Ito et al., 2020). Taking into consideration the safety of the noninvasive and radiation-free procedure, it is preferred over other ablative methods for patients with medically refractory ET (Health Quality Ontario, 2018).

However, the efficacy and adverse events (AEs) of MRgFUS among Chinese people have not yet been evaluated extensively. The clinical characteristics of ET might differ between Asians and Caucasians. Therefore, this study aimed to evaluate the outcome of MRgFUS thalamotomy among Taiwanese patients with refractory ET.

## Methods

### Patients

This retrospective study included patients with ET treated with focused ultrasound thalamotomy at an MRgFUS Center in Taiwan from 2017 to 2019. The procedure was performed by an experienced team. Patients with a neurodegenerative disorder such as Parkinson's disease (PD), cognitive impairment (Mini-Mental State Examination score of < 24), coagulopathy, and severe depression and those who were not followed up were excluded (Elias et al., 2016). Only patients with ET who underwent unilateral MRgFUS VIM thalamotomy with a follow-up duration of at least 6 months were included in the study. All patients underwent a preoperative brain CT scan, and data on cranial parameters including skull thickness, skull density ratio (SDR), and skull area were obtained. SDR was calculated by obtaining the average ratio of the cancellous to the cortical bone within the skull *via* a CT scan. Patients with an SDR of ≥0.3 were considered for MRgFUS treatment (Wintermark et al., 2014). The study was approved by the institutional review board of Show Chwan Memorial Hospital, Taiwan (IRB approval no.: 1090908).

### Magnetic Resonance-Guided Focused Ultrasound

Patients were treated with unilateral VIM thalamotomy with transcranial, noninvasive MRgFUS without general anesthesia. The head of the patient was shaved before treatment, and the scalp was examined for any scars or lesions that might interfere with ultrasound transcranial transmission. The MRI unit General Electric (Chicago, IL) 1.5T Optima MR450W was prepared. The stereotactic frame with a spherical coil and a water-cooling elastic diaphragm was placed over the head of the patient. The setup was connected to the ultrasound transducer (Exablate Neuro, InSightec, Tirat Carmel, Israel) coupled with a software unit.

**Figure 1:**
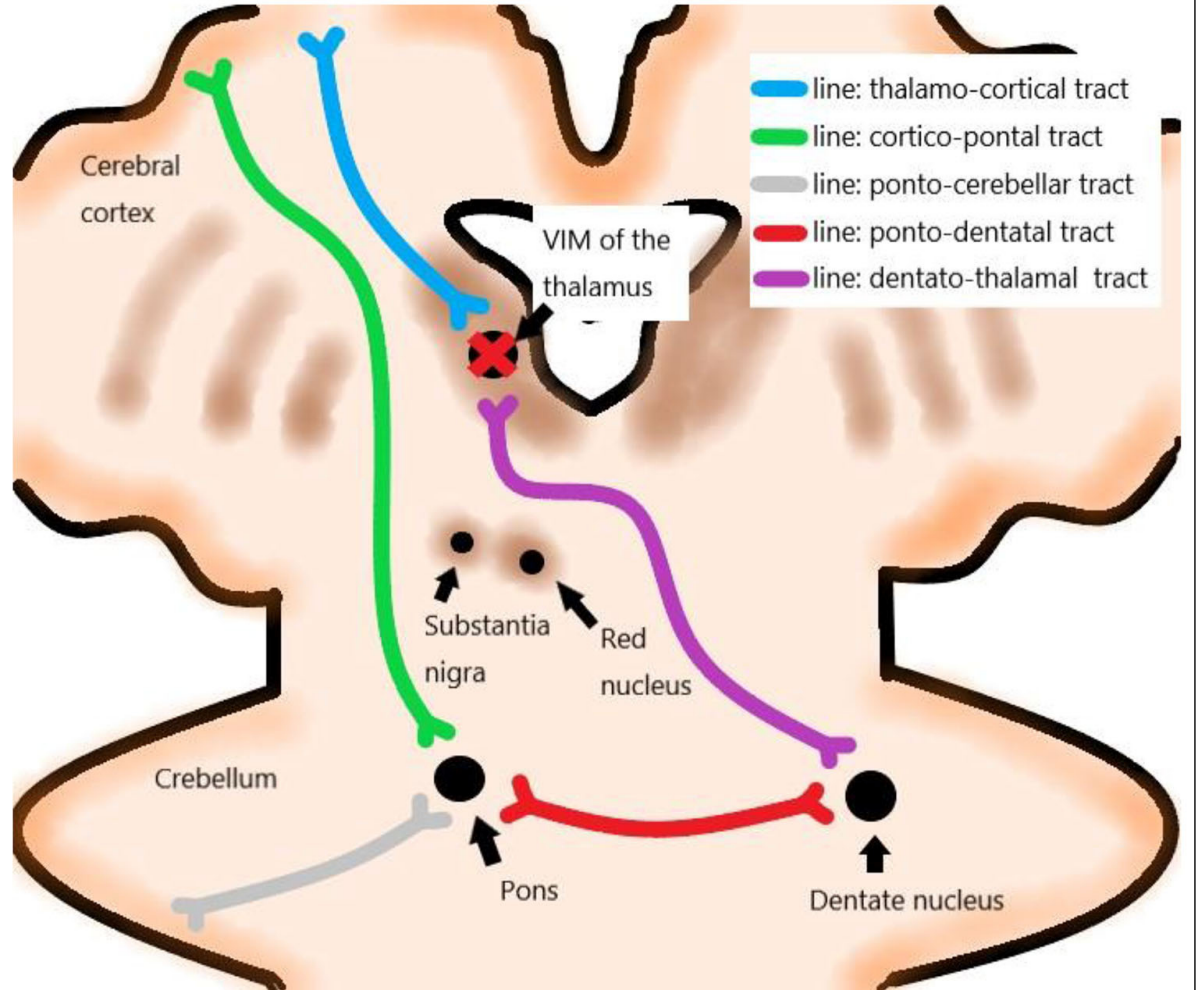
Networks correlated with essential tremor (ET). VIM: ventral intermediate nucleus.

The treatment targeted the VIM of the thalamus contralateral to the dominant side. VIM was localized from a functional viewpoint, and indirect targeting was performed based on data from stereotactic brain atlases and the previous experience of neurosurgeons (Figure 1) (Sharifi et al., 2014). The presumptive site of the VIM was localized at 14 mm from the midline or 11 mm from the lateral wall of the third ventricle and 1/3 from the posterior commissure (PC) on the intercommissural line or 7 mm anterior to the PC on MRI (Elias et al., 2013; Lipsman et al., 2013; Chang et al., 2015).

**Figure 2:**
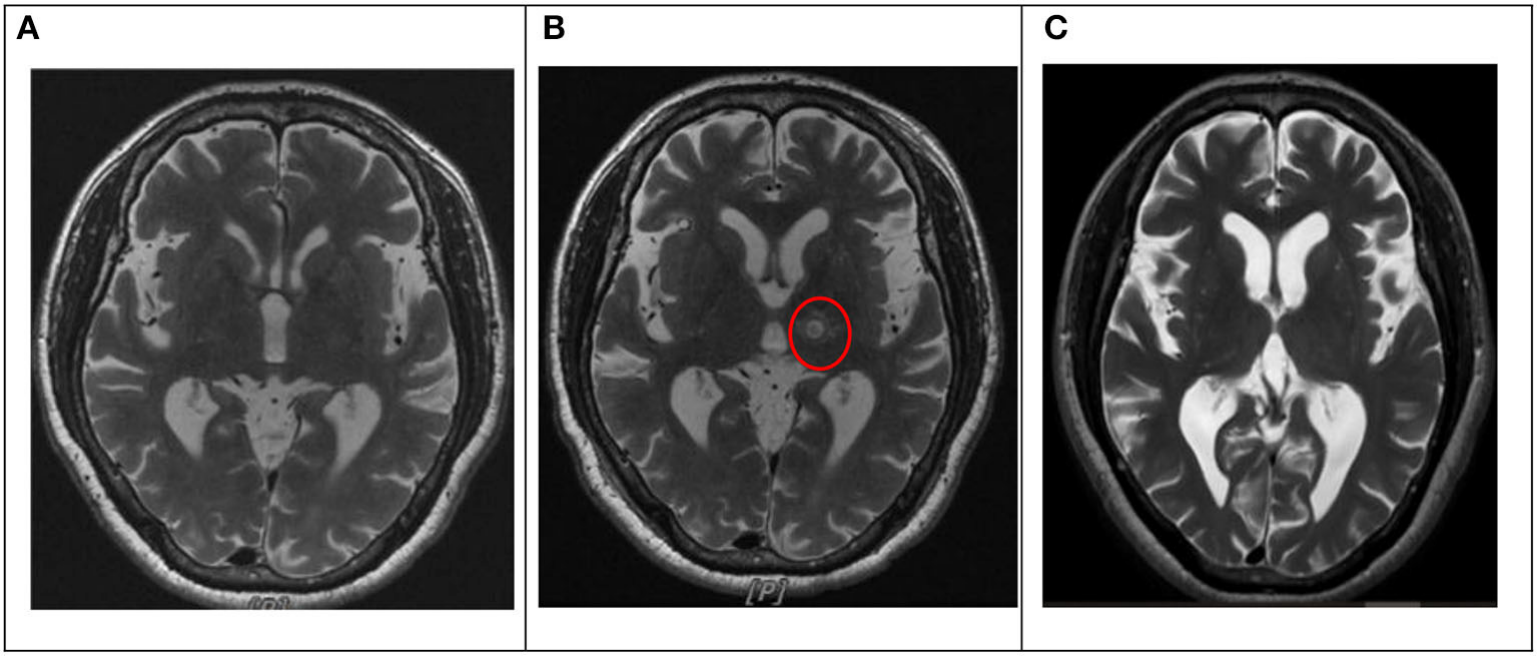
Lesion location on T2-weighted images before and after MRgFUS thalamotomy in one patient. **(A)** Pre-intervention. **(B)** Day 1. The target lesion was in the left VIM of the thalamus (red circle). **(C)** Six months. MRgFUS: magnetic resonance-guided focused ultrasound. VIM: ventral intermediate nucleus.

Patients underwent serial sonications for thalamic lesioning, starting with short and low energy, which produced focal heating of up to 44°C and progressed incrementally, thereby generating increasingly larger concentric lesions. The ultrasound energy was transmitted *via* 1,024 transducer elements, and it focused on the thalamus, creating the ablation at the focus by heating, which is tracked using MRI thermometry (Elias et al., 2013, 2016; Lipsman et al., 2013; Chang et al., 2015). The size and location of the lesion and clinical effects were continuously monitored. In particular, changes in tremors were closely monitored in the treated arm by asking patients for the presence of any AEs during treatment. Lesions progressively enlarged by increasing the temperature or duration of sonication until either tremor suppression is achieved or AEs are encountered. MRI was performed before treatment, immediately after the procedure, 1 day after the procedure, and 6 months after the procedure (Figure 2).

### Outcome Parameters

The Clinical Rating Scale for Tremor (CRST) is the standard assessment test for tremors in ET, and the CRST score was the primary outcome in this study. CRST has three parts (A, B, and C). Part A rates resting, postural, and action tremors for location and severity of tremors; part B rates hardwiring, drawing, and pouring of specific motor tasks; and part C rates speaking, eating, drinking, hygiene, dressing, writing, working, and social activities for functional disabilities. The score of each item ranges from 0 to 4 (total: 148), and a higher score indicates greater disease severity (Stacy et al., 2007). The dominant tremor score (maximum of 32) was defined as the sum of tremors in parts A and B for the treated hand. All patients were assessed using CRST by the same neurologists before treatment and were followed up after 1 week; 1, 3, and 6 months; and 1 and 2 years.

The incidence rate of AEs was considered the secondary outcome. AEs were classified as frame-, sonication-, and thalamotomy-related AEs for analysis purposes. Frame-related AEs included ptosis, pin-site bleeding, edema, and pain. Sonication-related AEs included vertigo or dizziness, headache, and nausea. Thalamotomy-related AEs included sensory-related events (numbness or paresthesia of various parts of body and taste disturbance), strength-related events (weakness of extremities), balance-related problems (dysmetria), and ataxia. Dysarthria and dysphagia were thalamotomy-related AEs (Elias et al., 2016). The AEs were recorded and followed up during the treatment day and after 1 day, 1 week, 3 and 6 months, and 1 and 2 years.

### Statistical Analysis

The total CRST scores and sub-scores during each follow-up were compared with the baseline scores using one-way repeated measures ANOVA with the least significant difference *post-hoc* analysis.

The relationships between skull factors and treatment parameters were evaluated *via* Pearson's correlation analysis and using the chi-square test. The skull factors included SDR, average skull thickness, skull volume, and skull area. Meanwhile, the treatment parameters included a total number of active elements (out of 1,024) in the spherical transducer, total energy transmitted, the maximum temperature reached upon completion of the procedure (Tmax), and sonication number and duration.

A *p*-value of<0.05 was considered statistically significant, and all analyses were conducted using the Statistical Package for the Social Sciences software version 24.0 (IBM Corp., Armonk, NY, the USA).

## Results

### Participants

In total, 48 patients, with a mean age of 59.2 ± 13.5 years and a mean duration of disease of 19.2 ± 13.6 years, were included in the analysis. Approximately 64.6% of patients were men, and 43.8% had a family history of ET. The dominant treated side was in the left (85.4%), and the mean SDR was 0.5 ± 0.1 (Table 1). The maximum, minimum, and median values of SDR were 0.7, 0.3, and 0.45. Twenty patients (25%) had SDRs of 0.40 or less.

**Table 1:** Baseline demographic and clinical characteristics.

**Variables**	**Mean ± SD**
Age, year	59.2 ± 13.5
Sex (male/female), *N* (%)	31 (64.6)/17 (35.4)
Treated side (left/right), *N* (%)	41 (85.4)/7 (14.6)
Family history (positive/negative/uncertain), *N* (%)	21 (43.8)/20 (41.7)/7 (14.5)
Disease duration, year	19.2 ± 13.6
SDR	0.5 ± 0.1
Average skull thickness, cm	7.1 ± 1.0
Skull area, cm^2^	366.2 ± 24.9
Average IA, degree	12.7 ± 1.1
No. of IA, < 20	892.2 ± 62.3
No. of IA, < 25	969.6 ± 34.9
Active elements	985.4 ± 32.0
No. of sonications	10.0 ± 2.6
No. of sonications, ≥ 50°C	4.6 ± 2.2
T max, °C	57.0 ± 2.4
Average total energy, J	97908.6 ± 60052.6
Average maximum energy, J	19710.5 ± 8624.9

*SD, Standard Deviation; SDR, Skull Density Ratio; IA, Incident Angle; T max, Maximum Temperature reached after completion of the procedure*.

### Tremor

**Figure 3:**
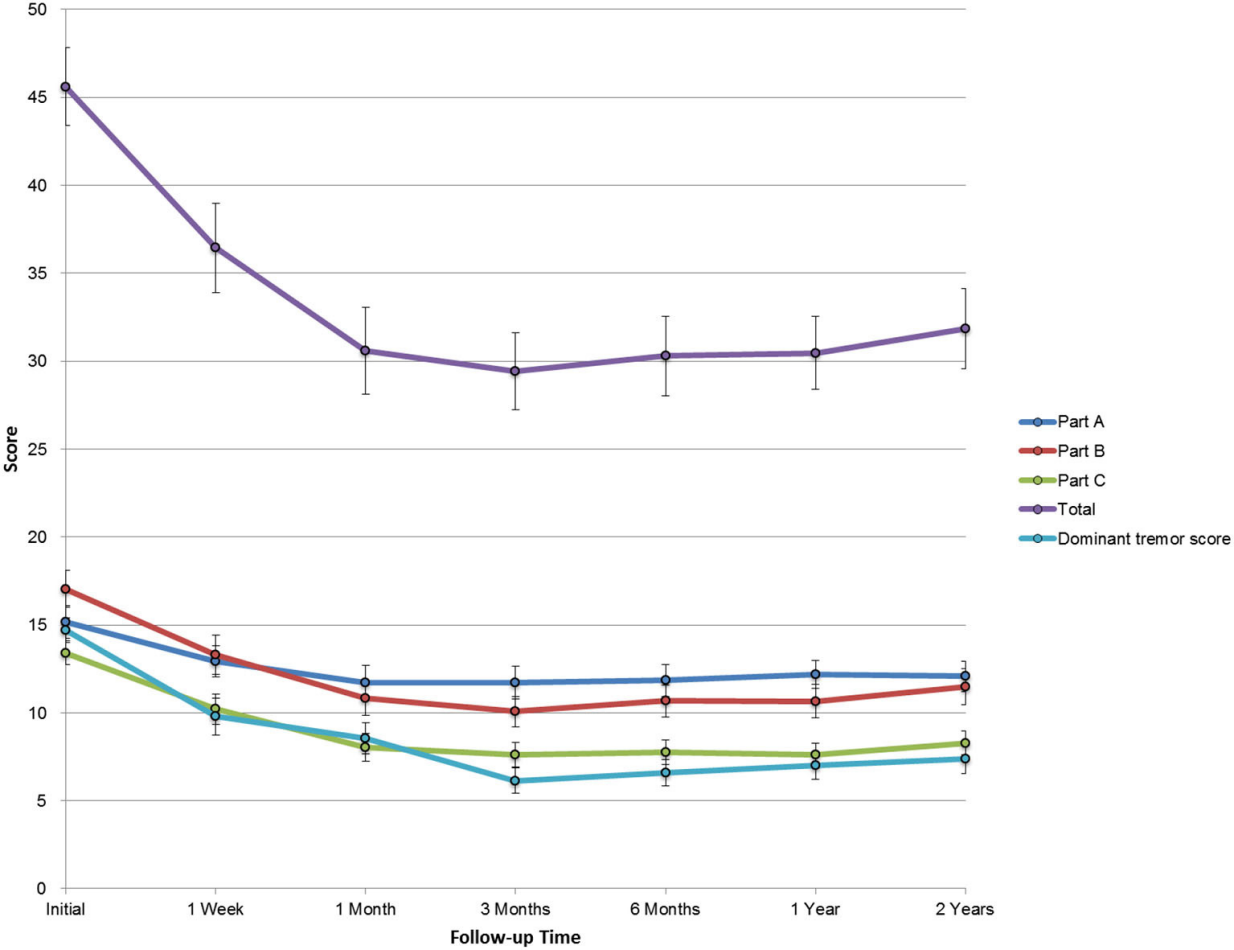
The trend in sub-scores during the 2-year follow-up. CRST: Clinical Rating Scale for Tremor. Error bars were plotted with SE.

The total CRST score at baseline (parts A, B, and C) was 45.6 ± 15.4, and it reduced to 36.4 ± 17.6, 30.6 ± 17.1, 29.4 ± 15.1, 30.3 ± 15.7, 30.5 ± 14.4, and 31.9 ± 15.8 after 1 week, 1 month, 3 months, 6 months, 1 year, and 2 years, respectively (*p* < 0.001). The baseline dominant tremor score was 14.7 ± 4.9, and it reduced to 9.8 ± 7.3, 8.6 ± 6.2, 6.2 ± 5.0, 6.6 ± 5.3, 7.0 ± 5.5, and 7.4 ± 5.8 after 1 week, 1 month, 3 months, 6 months, 1 year, and 2 years, respectively (*p* < 0.001). The total disability score (part C) significantly improved from 13.4 ± 4.6 to 10.2 ± 5.9, 8.0 ± 5.6, 7.6 ± 4.8, 7.8 ± 5.0, 7.6 ± 4.6, and 8.3 ± 5.4 after 1 week, 1 month, 3 months, 6 months, 1 year, and 2 years, respectively (*p* < 0.001). Furthermore, parts A and B were decreased at any posttreatment stage (*p* < 0.001, Table 2). The trends in the total CRST scores and sub-scores during follow-up are shown in Figure 3.

**Table 2:** Total CRST scores and sub-scores during each follow-up (*N* = 48).

**Evaluation time**	**Part A**	**Part B**	**Part C**	**Total**	**Dominant tremor score**
Baseline	15.2 ± 6.5	17.0 ± 7.2	13.4 ± 4.6	45.6 ± 15.4	14.7 ± 4.9
1 week	12.9 ± 6.2^*^	13.3 ± 7.8^*^	10.2 ± 5.9^*^	36.4 ± 17.6^*^	9.8 ± 7.3^*^
1 month	11.7 ± 6.5^*^	10.8 ± 6.9^*^	8.0 ± 5.6^*^	30.6 ± 17.1^*^	8.6 ± 6.2^*^
3 months	11.7 ± 6.4^*^	10.1 ± 6.0^*^	7.6 ± 4.8^*^	29.4 ± 15.1^*^	6.2 ± 5.0^*^
6 months	11.9 ± 6.3^*^	10.7 ± 6.3^*^	7.8 ± 5.0^*^	30.3 ± 15.7^*^	6.6 ± 5.3^*^
1 year	12.2 ± 5.4^*^	10.7 ± 6.8^*^	7.6 ± 4.6^*^	30.5 ± 14.4^*^	7.0 ± 5.5^*^
2 years	12.1 ± 6.0^*^	11.5 ± 7.2^*^	8.3 ± 4.8^*^	31.9 ± 15.8^*^	7.4 ± 5.8^*^

*CRST, Clinical Rating Scale for Tremor*.

* *Significant level at p < 0.001 compared with the initial score. Data were presented as mean ± SD. Analyses were performed using one-way repeated measures ANOVA with the least significant difference post-hoc analysis*.

### Adverse Events

Frame-related events were not observed after 1 month. Sonication-related AEs were predominant on the day of treatment. However, they were transient and subsided after 1 week in all patients. Ataxia was the major thalamotomy-related AE. The incidence rates were 83.3, 41.7, 10.4, 6.3, and 4.2% after 1 day, 1 week, 1 month, 3 months, and 6 months, respectively. Complete disappearance was observed after the 6-month follow-up. Approximately 8% of patients complained of sensory-related AEs, including numbness of the tongue and/or fingertip, which persisted during the 2-year follow-up. However, worsening or new AEs were not observed during the 2-year follow-up (Table 3).

**Table 3:** Adverse events after MRgFUS thalamotomy (*N* = 48).

**Subgroups**	**Frame-related AEs**	**Sonication-related AEs**	**Thalamotomy-related AEs**				**Others**
			**Sensory-related**	**Strength-related**	**Balance-related**	**Dysarthria and dysphagia**	
0 day	0 (0)	27 (56.3)	2 (4.2)	0 (0)	0 (0)	0 (0)	3 (6.3)
1 day	0 (0)	2 (4.2)	4 (8.3)	2 (4.2)	40 (83.3)	1 (2.1)	3 (6.3)
1 week	1 (2.1)	1 (2.1)	3 (6.3)	3 (6.3)	20 (41.7)	1 (2.1)	10 (20.8)
1 month	0 (0)	0 (0)	5 (10.4)	3 (6.3)	5 (10.4)	2 (4.2)	7 (14.6)
3 months	0 (0)	0 (0)	4 (8.3)	2 (4.2)	3 (6.3)	2 (4.2)	8 (16.7)
6 months	0 (0)	0 (0)	3 (6.3)	2 (4.2)	2 (4.2)	0 (0)	5 (10.4)
1 year	0 (0)	0 (0)	3 (6.3)	2 (4.2)	0 (0)	0 (0)	3 (6.3)
2 years	0 (0)	0 (0)	4 (8.3)	0 (0)	0 (0)	0 (0)	0 (0)

*AEs, Adverse Events; MRgFUS, Magnetic Resonance-guided Focused Ultrasound*.

*Data were presented as numbers (%)*.

## Discussion

Pharmacological agents such as propranolol and primidone are the primary choice of most physicians as they are recommended by the American Academy of Neurology (AAN) (Zesiewicz et al., 2011). However, even if these agents do not cause AEs, the efficacy rate is only 50–70% (Haubenberger and Hallett, 2018). Recent studies have shown that propranolol may be associated with an increased risk of PD (Mittal et al., 2017; Gronich et al., 2018; Hopfner et al., 2019). Surgical interventions including DBS and MRgFUS are recommended for medically refractory cases; MRgFUS is a novel technique that is less invasive than DBS (Shanker, 2019). Hence, in Taiwan, patients with refractory ET are more likely to be treated with MRgFUS than DBS.

In this retrospective study including 48 patients with drug-refractory ET, MRgFUS thalamotomy significantly reduced hand tremor immediately, and the effect persisted until the 2-year follow-up period. The total CRST scores improved by 35.3, 33.5, and 32.5% after 3 months, 1 year, and 2 years, respectively. The dominant tremor scores improved by 64.0, 60.1, and 58.2% after 3 months, 1 year, and 2 years, respectively. The outcome in this study was not worse than that in previous larger studies (*N*, ≥ 30; Table 4) (Elias et al., 2016; Chang et al., 2018; Meng et al., 2018). After the achievement of postoperative reduction, the beneficial effect was maintained until the 2-year follow-up period.

**Table 4:** Comparison of outcomes between the current and previous studies with a larger sample size (*N* ≥ 30).

**Author, year**	***N***	**Rate of improvement rate in dominant tremor scores**		
		**6 months**	**1 year**	**2 years**
The study	48	55.1%	52.4%	49.7%
Meng et al., 2018	37	No data	42.4%	43.4%
Chang et al., 2018	76	56.6%	55.0%	55.6%
Elias et al., 2016	56	44.0%	39.8%	No data

The AEs were mostly transient and mild. The incidence rates of sensory deficit and imbalance after the intervention ranged from 13% to 19% (Park et al., 2019; Sinai et al., 2019). In this study, the frame- and sonication-related AEs disappeared within 1 week. Vasogenic edema over the outer layer, which was treated with dexamethasone, was correlated with symptom relief. However, 6.3 and 8.3% of patients experienced sensory problems that lasted for 1 and 2 years, respectively. The incidence rates of long-term thalamotomy-related AEs in Chinese individuals were not higher than that in Western individuals according to previous reports (Elias et al., 2016; Chang et al., 2018; Meng et al., 2018). Interestingly, during follow-up, diminished leukoaraiosis was observed on **T2**-weighted-fluid-attenuated inversion recovery (T2 FLAIR), and the accumulation of hemosiderin was observed on susceptibility-weighted imaging (SWI) series (Figure 2). We hypothesized that this might be the difference between functional lesioning by focused ultrasound and traditional lesioning by radiofrequency ablation. The accumulation of hemosiderin deposits may influence the connection of the dentatorubrothalamic tract. In addition, the SDR values were proportionally correlated with the incidence of AEs. In addition, while a blood-oxygen-level-dependent (BOLD) signal in resting-state functional MRI (fMRI) analysis reveals a focal lesion in the MRgFUS VIM thalamotomy that results in symptom-related, long-term alterations in the effective connectivity of the dentate nucleus in the motor circuit, a similar experiment also could be conducted to explore the effective connectivity difference among AEs (Park et al., 2017).

The proportion of men and women affected by ET is equal. However, hand tremor was more severe in men than in women, and head and voice tremors were more severe in women than in men (Shanker, 2019). MRgFUS thalamotomy is more promising in controlling peripheral tremors, and this may be the reason why more male patients received this treatment. Identifying brain targets for resolving head and voice tremors under safety procedures will be an important issue in future studies.

From a technical viewpoint, the energy efficiency is lower in the low SDR group than in the high SDR group (Chang et al., 2016, 2020). Patients can be at risk for long treatment duration due to different kinds of discomforts, including the tightness and pressure from the silicon membrane attached to the head of the patient, the headache caused by cold water between the transducer and the membrane, the pain caused by the pins of the stereotactic frame, and deep vein thrombosis. Based on a balance between clinical benefits and risks, patients with a high SDR (>0.45) must be selected, which is based on the recommendation of the FDA (U.S. Food Drug Administration, 2020a,b). However, this study had shown that there were no significant differences in terms of clinical outcome and safety profile between patients with a low SDR (0.3–0.4) and those with a high SDR (>0.4). Similar reports of Japan and Korea also showed that patients with low SDR can be successfully treated (Chang et al., 2019; Yamamoto et al., 2019). As reported previously, 50% of Chinese and 78.6% of Japanese patients had SDRs of 0.40 or less (Yamamoto et al., 2019; Tsai et al., 2021). Increasing the efficacy of ablation in treating such patients with low SDR is an important issue. During the preparation of the patient, we attempted to enlarge the skull surface, if possible, by placing the membrane as inferior as possible to prevent exceeding the safety limit of 100 J/cm^2^. Meanwhile, the membrane fold could be excluded from the region of focused ultrasound penetration. Hence, to achieve better focus, the number of active elements can be increased (Yamamoto et al., 2020).

In this study, we used the conventional stereotactic coordinate to determine the initial target location for the VIM thalamotomy. The tremor control (Figure 3) and the reported AE (Table 3) indicated that this targeting strategy leads to similar clinical outcomes as the other reports (Elias et al., 2016; Chang et al., 2018; Meng et al., 2018). Recently, the diffusion-weighted MRI was reported and reviewed (Lehman et al., 2020) to facilitate the targeting of the dentatorubrothalamic tract in order to improve symptom control (Chazen et al., 2018) or avoid the potential adverse effects (Krishna et al., 2019). Although there is an existing limitation of diffusion-tensor imaging (DTI) regarding the image distortion and the manual error of this seed-based tractography targeting, this advanced targeting strategy is still promising and could be a valuable reference for this MRgFUS procedure.

Lesion size is associated with the clinical efficacy of MRgFUS thalamotomy in ET (Federau et al., 2018; Harary et al., 2018). At the time of treatment, 3–6 sonications are normally applied to align the center of the hot spot to the selected target; then, the following sonications are used to stimulate the patient, achieve the therapeutic temperature, and consolidate the lesion if there is any. Empirically, the energy efficiency can be lower, and the perifocal edema can be larger if the number of sonications is higher. Hence, instead of moving around the target region to identify a more effective location, a small number of sonications were used, and the treatment was completed to address the aforementioned issues. To date, a high temperature is utilized to create the lesion and control the tremor.

This study was not a randomized controlled trial to ensure efficacy and safety (Elias et al., 2016) but is based on the real-world data like other similar research for safety, effectiveness, and long-term outcomes (Chang et al., 2018; Katkade et al., 2018; Meng et al., 2018; Park et al., 2019; Sinai et al., 2019). It had some limitations. First, retrospective data were collected only for up to 2 years. Hence, a longer follow-up duration is necessary. Second, further analyses of lesions and procedures, which are important in improving the efficacy of treatment and reducing the incidence of AEs, were not performed. Third, the Quality of Life in Essential Tremor Questionnaire (QUEST) was not used. Hence, the quality of life among patients with ET was not evaluated.

In conclusion, the incidence of tremor and disability decreased after MRgFUS thalamotomy among patients with drug-refractory ET. The outcome of this study was similar to that of previous studies, which had <2 years of follow-up. MRgFUS can be performed successfully for patients with normal and low SDR. Therefore, this study suggests the possibility that lowering the cutoff value of SDR (≥0.3) may result in more patients being treated with this nonsurgical and nonionizing intervention.

## Data Availability Statement

The original contributions presented in the study are included in the article/supplementary material, further inquiries can be directed to the corresponding authors.

## Ethics Statement

The studies involving human participants were reviewed and approved by Institutional Review Board of Show Chwan Memorial Hospital. The patients/participants provided their written informed consent to participate in this study.

## Author Contributions

PW and WL performed the literature search and wrote the results. KHL and H-CL collected and edited the clinical data. M-TL participated in data analysis. KW-KT wrote the discussion section. P-YC revised the manuscript. W-CC revised and drafted the manuscript. C-YW contributed to the revisions and final draft of the manuscript. TT provided clinical recommendations. All authors have read and approved the final version of the manuscript.

## Conflict of Interest

KW-KT was employed by company Insightec Ltd. The authors declare that part of this study received funding from Insightec Ltd. under the ET002J protocol. The funder had involvement with the preparation of the manuscript.

## References

[B1] Blomstedt P. Hariz M.I. (2005). Hardware-related complications of deep brain stimulation: a ten year experience. Acta Neurochir. 147, 1061–1064. discussion 1064. 10.1007/s00701-005-0576-5 16041470

[B2] Chang J.W. Park C.K. Lipsman N. Schwartz M.L. Ghanouni P. Henderson J.M.. (2018). A prospective trial of magnetic resonance-guided focused ultrasound thalamotomy for essential tremor: results at the 2-year follow-up. Ann. Neurol. 83, 107–114. 10.1002/ana.25126 29265546

[B3] Chang K.W. Park Y.S. Chang J.W. (2019). Skull factors affecting outcomes of magnetic resonance-guided focused ultrasound for patients with essential tremor. Yonsei Med. J. 60, 768–773. 10.3349/ymj.2019.60.8.768 31347332 PMC6660436

[B4] Chang K.W. Rachmilevitch I. Chang W.S. Jung H.H. Zadicario E. Prus O.. (2020). Safety and efficacy of magnetic resonance-guided focused ultrasound surgery with autofocusing echo imaging. Front. Neurosci. 14:592763. 10.3389/fnins.2020.592763 33510610 PMC7835836

[B5] Chang W.S. Jung H.H. Kweon E.J. Zadicario E. Rachmilevitch I. Chang J.W. (2015). Unilateral magnetic resonance guided focused ultrasound thalamotomy for essential tremor: practices and clinicoradiological outcomes. J. Neurol. Neurosurg. Psychiatry 86, 257–264. 10.1136/jnnp-2014-307642 24876191

[B6] Chang W.S. Jung H.H. Zadicario E. Rachmilevitch I. Tlusty T. Vitek S.. (2016). Factors associated with successful magnetic resonance-guided focused ultrasound treatment: efficiency of acoustic energy delivery through the skull. J. Neurosurg. 124, 411–416. 10.3171/2015.3.JNS142592 26361280

[B7] Chazen J.L. Sarva H. Stieg P.E. Min R.J. Ballon D.J. Pryor K.O.. (2018). Clinical improvement associated with targeted interruption of the cerebellothalamic tract following MR-guided focused ultrasound for essential tremor. J. Neurosurg. 129, 315–323. 10.3171/2017.4.JNS162803 29053074

[B8] Diaz N.L. Louis E.D. (2010). Survey of medication usage patterns among essential tremor patients: movement disorder specialists vs. general neurologists. Parkinsonism Relat. Disord. 16, 604–607. 10.1016/j.parkreldis.2010.07.011 20691629 PMC2963696

[B9] Elias W.J. Huss D. Voss T. Loomba J. Khaled M. Zadicario E.. (2013). A pilot study of focused ultrasound thalamotomy for essential tremor. N. Engl. J. Med. 369, 640–648. 10.1056/NEJMoa1300962 23944301

[B10] Elias W.J. Lipsman N. Ondo W.G. Ghanouni P. Kim Y.G. Lee W.. (2016). A randomized trial of focused ultrasound thalamotomy for essential tremor. N. Engl. J. Med. 375, 730–739. 10.1056/NEJMoa1600159 27557301

[B11] Engel K. Huckhagel T. Gulberti A. Pötter-Nerger M. Vettorazzi E. Hidding U.. (2018). Towards unambiguous reporting of complications related to deep brain stimulation surgery: a retrospective single-center analysis and systematic review of the literature. PLoS ONE 13:e0198529. 10.1371/journal.pone.0198529 30071021 PMC6071984

[B12] Federau C. Goubran M. Rosenberg J. Henderson J. Halpern C.H. Santini V.. (2018). Transcranial MRI-guided high-intensity focused ultrasound for treatment of essential tremor: a pilot study on the correlation between lesion size, lesion location, thermal dose, and clinical outcome. J. Magn. Reson. Imag. 48, 58–65. 10.1002/jmri.25878 29076274

[B13] Findley L.J. Cleeves L. Calzetti S. (1985). Primidone in essential tremor of the hands and head: a double blind controlled clinical study. J. Neurol. Neurosurg. Psychiatry 48, 911–915. 10.1136/jnnp.48.9.911 3900296 PMC1028493

[B14] Gronich N. Abernethy D.R. Auriel E. Lavi I. Rennert G. Saliba W. (2018). β2-adrenoceptor agonists and antagonists and risk of Parkinson's disease. Mov. Disord. 33, 1465–1471. 10.1002/mds.108 30311974

[B15] Harary M. Essayed W.I. Valdes P.A. McDannold N. Cosgrove G.R. (2018). Volumetric analysis of magnetic resonance-guided focused ultrasound thalamotomy lesions. Neurosurg. Focus 44:E6. 10.3171/2017.11.FOCUS17587 29385921

[B16] Haubenberger D. Hallett M. (2018). Essential Tremor. N. Engl. J. Med. 378, 1802–1810. 10.1056/NEJMcp1707928 29742376

[B17] Health Quality Ontario (2018). Magnetic resonance-guided focused ultrasound neurosurgery for essential tremor: a health technology assessment. Ont. Health Technol. Assess. Ser. 18, 1–141. PMC5963668 29805721

[B18] Hopfner F. Wod M. Höglinger G.U. Blaabjerg M. Rösler T.W. Kuhlenbäumer G.. (2019). Use of β2-adrenoreceptor agonist and antagonist drugs and risk of Parkinson disease. Neurology 93, e135–e142. 10.1212/WNL.0000000000007694 31127070

[B19] Ito H. Yamamoto K. Fukutake S. Odo T. Kamei T. (2020). Two-year follow-up results of magnetic resonance imaging-guided focused ultrasound unilateral thalamotomy for medication-refractory essential tremor. Intern. Med. 59, 2481–2483. 10.2169/internalmedicine.4360-19 32641664 PMC7662049

[B20] Katkade V.B. Sanders K.N. Zou K.H. (2018). Real world data: an opportunity to supplement existing evidence for the use of long-established medicines in health care decision making. J. Multidiscip. Healthcare 11, 295–304. 10.2147/JMDH.S160029 29997436 PMC6033114

[B21] Koller W.C. Vetere-Overfield B. (1989). Acute and chronic effects of propranolol and primidone in essential tremor. Neurology 39, 1587–1588. 10.1212/WNL.39.12.1587 2586774

[B22] Krishna V. Sammartino F. Agrawal P. Changizi B.K. Bourekas E. Knopp M.V.. (2019). Prospective tractography-based targeting for improved safety of focused ultrasound thalamotomy. Neurosurgery 84, 160–168. 10.1093/neuros/nyy020 29579287

[B23] Lehman V.T. Lee K.H. Klassen B.T. Blezek D.J. Goyal A. Shah B.R.. (2020). MRI and tractography techniques to localize the ventral intermediate nucleus and dentatorubrothalamic tract for deep brain stimulation and MR-guided focused ultrasound: a narrative review and update. Neurosurg. Focus 49, E8. 10.3171/2020.4.FOCUS20170 32610293 PMC8032505

[B24] Lipsman N. Schwartz M.L. Huang Y. Lee L. Sankar T. Chapman M.. (2013). MR-guided focused ultrasound thalamotomy for essential tremor: a proof-of-concept study. Lancet Neurol. 12, 462–468. 10.1016/S1474-4422(13)70048-6 23523144

[B25] Louis E.D. Ferreira J.J. (2010). How common is the most common adult movement disorder? Update on the worldwide prevalence of essential tremor. Mov. Disord. 25, 534–541. 10.1002/mds.22838 20175185

[B26] Louis E.D. Joyce J.L. Cosentino S. (2019). Mind the gaps: what we don't know about cognitive impairment in essential tremor. Parkinsonism Relat. Disord. 63, 10–19. 10.1016/j.parkreldis.2019.02.038 30876840 PMC6682425

[B27] Meng Y. Solomon B. Boutet A. Llinas M. Scantlebury N. Huang Y.. (2018). Magnetic resonance-guided focused ultrasound thalamotomy for treatment of essential tremor: a 2-year outcome study. Mov. Disord. 33, 1647–1650. 10.1002/mds.99 30288794

[B28] Mittal S. Bjørnevik K. Im D.S. Flierl A. Dong X. Locascio J.J.. (2017). β2-Adrenoreceptor is a regulator of the α-synuclein gene driving risk of Parkinson's disease. Science 357, 891–898. 10.1126/science.aaf3934 28860381 PMC5761666

[B29] Park H.J. Pae C. Friston K. Jang C. Razi A. Zeidman P.. (2017). Hierarchical dynamic causal modeling of resting-state fMRI reveals longitudinal changes in effective connectivity in the motor system after thalamotomy for essential tremor. Front. Neurol. 8:346. 10.3389/fneur.2017.00346 28775707 PMC5517411

[B30] Park Y.S. Jung N.Y. Na Y.C. Chang J.W. (2019). Four-year follow-up results of magnetic resonance-guided focused ultrasound thalamotomy for essential tremor. Mov. Disord. 34, 727–734. 10.1002/mds.27637 30759322

[B31] Shanker V. (2019). Essential tremor: diagnosis and management. BMJ 366:l4485. 10.1136/bmj.l4485 31383632

[B32] Sharifi S. Nederveen A.J. Booij J. van Rootselaar A.F. (2014). Neuroimaging essentials in essential tremor: a systematic review. Neuroimage Clin. 5, 217–231. 10.1016/j.nicl.2014.05.003 25068111 PMC4110352

[B33] Sinai A. Nassar M. Eran A. Constantinescu M. Zaaroor M. Sprecher E.. (2019). Magnetic resonance-guided focused ultrasound thalamotomy for essential tremor: a 5-year single-center experience. J. Neurosurg. 133, 417–424. 10.3171/2019.3.JNS19466 31277064

[B34] Stacy M.A. Elble R.J. Ondo W.G. Wu S.C. Hulihan J. (2007). Assessment of interrater and intrarater reliability of the Fahn–Tolosa–Marin Tremor Rating Scale in essential tremor. Mov. Disord. 22, 833–838. 10.1002/mds.21412 17343274

[B35] SunH.SunF.ZhangX.Q.FangX.H.ChanP. (2020). The prevalence and clinical characteristics of essential tremor in elderly chineses: a population-based study. J. Nutr. Health Aging 24, 1061–1065. 10.1007/s12603-020-1472-733244561

[B36] Tsai K.W. Chen J.C. Lai H.C. Chang W.C. Taira T. Chang J.W.. (2021). The distribution of skull score and skull density ratio in tremor patients for mr-guided focused ultrasound thalamotomy. Front. Neurosci. 15:612940. 10.3389/fnins.2021.612940 34079434 PMC8165389

[B37] U.S. Food Drug Administration (2020a). InSightec ExAb¬late® System information for prescribers [Online]. Available online at: https://www.accessdata.fda.gov/cdrh_docs/pdf15/P150038C.pdf (Accessed Jun 7, 2020).

[B38] U.S. Food Drug Administration (2020b). Summary of Safety and Effectiveness Data [Online]. Available online at: https:// www.accessdata.fda.gov/cdrh_docs/pdf15/P150038B.pdf (Accessed Jun 7, 2020).

[B39] Wintermark M. Druzgal J. Huss D.S. Khaled M.A. Monteith S. Raghavan P.. (2014). Imaging findings in MR imaging-guided focused ultrasound treatment for patients with essential tremor. AJNR Am. J. Neuroradiol. 35, 891–896. 10.3174/ajnr.A3808 24371027 PMC7964546

[B40] Yamamoto K. Ito H. Fukutake S. Kamei T. Yamaguchi T. Taira T. (2019). Ventralis intermedius thalamotomy with focused ultrasound for patients with low skull density ratio. Mov. Disord. 34, 1239–1240. 10.1002/mds.27726 31136679

[B41] Yamamoto K. Ito H. Fukutake S. Odo T. Kamei T. Yamaguchi T.. (2020). Factors associated with heating efficiency in transcranial focused ultrasound therapy. Neurol. Med. Chir. (Tokyo) 60, 594–599. 10.2176/nmc.oa.2020-0225 33162467 PMC7803702

[B42] Zesiewicz T.A. Elble R.J. Louis E.D. Gronseth G.S. Ondo W.G. Dewey R.B. Jr.. (2011). Evidence-based guideline update: treatment of essential tremor: report of the Quality Standards subcommittee of the American Academy of Neurology. Neurology 77, 1752–1755. 10.1212/WNL.0b013e318236f0fd 22013182 PMC3208950

